# Personal Pervasive Environments: Practice and Experience

**DOI:** 10.3390/s120607109

**Published:** 2012-05-29

**Authors:** Francisco J. Ballesteros, Gorka Guardiola, Enrique Soriano

**Affiliations:** Laboratorio de Sistemas, Universidad Rey Juan Carlos, E-28943 Madrid, Spain; E-Mail: nemo@lsub.org

**Keywords:** operating systems, middleware, pervasive computing, AmI

## Abstract

In this paper we present our experience designing and developing two different systems to enable personal pervasive computing environments, Plan B and the Octopus. These systems were fully implemented and have been used on a daily basis for years. Both are based on synthetic (virtual) file system interfaces and provide mechanisms to adapt to changes in the context and reconfigure the system to support pervasive applications. We also present the main differences between them, focusing on architectural and reconfiguration aspects. Finally, we analyze the pitfalls and successes of both systems and review the lessons we learned while designing, developing, and using them.

## 1. Introduction

Understanding the issues present in system software design and development requires to implement different approaches and evaluate their effectiveness in practice, by using them on a daily basis for some time. We applied this method to evaluate two different operating systems aimed at enabling *personal pervasive environments*, Plan B and the Octopus. For a more detailed and formal description of these systems refer to [1–4].

The goal of a *personal pervasive environment* is to provide a single system image: a per-user virtual computer composed by the personal, disperse resources, including data, devices, and applications, and other services imported from a smart space, such as a context data infrastructure, storage, sensors, actuators, computing facilities, *etc.*

Both Plan B and the Octopus enable this kind of environments. In order to do that, they provide services and mechanisms for event delivery, reconfiguration and adaptation, service discovery, protection, *etc.* Plan B and the Octopus follow different design and implementation approaches. On the other hand, these systems are similar in one design principle: both use synthetic (virtual) files as the main interface. Resources are represented by the servers as tiny synthetic file systems exported through the network, and are accessed by the client through the file handling system calls (mostly *open, read, write*, and *close*).

The file system abstraction provides a portable distributed representation of the components of the system. Nevertheless, the system needs a mechanism to reconfigure and adapt to changes in the environment (e.g., changes in the user context). This mechanism must be in charge of changing the namespace, that is, remapping the correspondence between names (e.g., paths in the file system) and resources (e.g., an X10 actuator). In our case, this correspondence is managed by a *mount table*, which binds different files served by different file servers in a unique space. In this respect, Plan B and the Octopus follow different architectures and therefore have different compromises in the design.

This paper aims to:

- Compare the different architectures and their approaches to adapt the namespaces to changes in the context.
- Describe the general aspects, components, and applications that turned out to be successful and the ones that did not work well or were not very useful in practice.
- Present the lessons learned and the experience after designing, developing, and using Plan B and the Octopus. As expected, the latter solved many of the problems raised while implementing the former, but it also raised new issues and pitfalls.

## 2. From Plan B to the Octopus

Next, we briefly describe some key aspects of the two systems. First we introduce their general architecture. Then, we explain how the different approaches influence their adaptation and configuration mechanisms. In short, the main differences are:

- Decentralized (Plan B is Peer-to-Peer) *vs.* centralized (Octopus).
- Independent peer namespace (Plan B) *vs.* global namespace (Octopus).
- Native OS *vs.* hosted OS.

### 2.1. Architecture

Plan B is an operating system that runs on bare hardware. The architecture of the system is Peer-to-Peer. Each machine running Plan B on behalf of the user is a *peer*. A peer exports its own resources and imports resources from known peers according to defined constrains and context information. The peers announce their resources using a discovery protocol, and they are in charge of keeping track of the available resources that come and go. A peer manages its own namespace and binds concrete resources at well-known points of its tree hierarchy. It is also in charge of multiplexing the resources it imports, depending on the context data and constraints set by the user.

**Figure 1.**
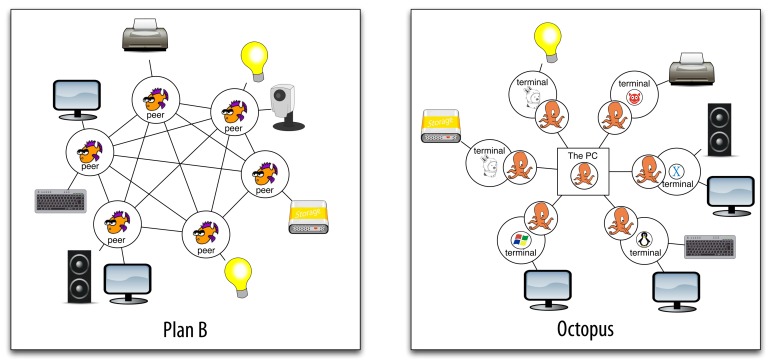
The architecture of Plan B (**left**) and the Octopus (**right**).

The Octopus follows a different approach. The Octopus is an operating system that can run hosted on a conventional system. The architecture is based on a central service provided by the Cloud or a centralized node. It centralizes the control and provides a global namespace for all of the client machines. When a client (terminal) boots, it is registered in the central control service. Then, the local resources exported by the terminal are aggregated into the global namespace. The terminal also imports the global namespace, so it is able to reach any resource of the system. Resources are made available for the host system (and its applications) through the global namespace. The central control service is in charge of multiplexing the resources according to context data, providing a default resource of each class (the same default resources for all the terminals of the user). Figure 1 depicts two example scenarios to highlight the differences between the architectures of these systems.

### 2.2. Namespaces and Reconfiguration Mechanisms

**Figure 2.**
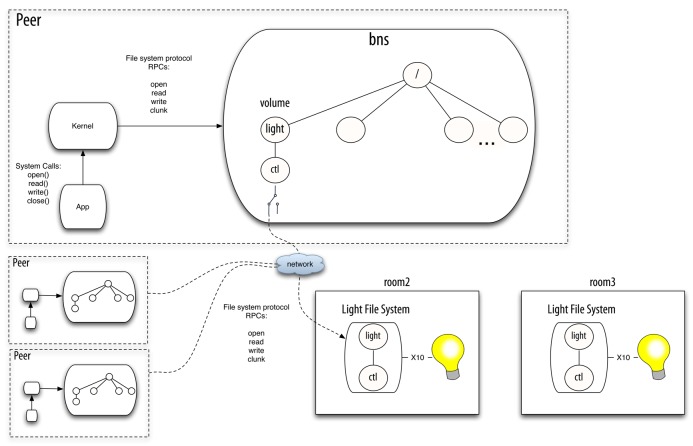
An example scenario with bns.

Plan B [1,2] manages the namespace and adapts to changes using a component called bns. There is one instance of this userspace program running in each Plan B machine (*i.e.*, each peer). Bns is a network file system protocol multiplexer. It receives 9P (the network file system protocol of Plan 9) operations and sends them to the selected file server. When the user mounts a resource (in Plan B this is called a *volume*), she provides a description of its properties (class of resource, location, and so on). Bns is in charge of attaching to the remote file system and forwarding the operations to it. It is also in charge of changing which volume is mapped to which specific file server. To do this, bns matches the properties specified by the user against the properties of the available resources (exported by peers). Bns learns about available resources through either a discovery protocol or direct commands from the user. The application performs regular system calls to operate on files. The Plan B kernel (in the same way the Plan 9 kernel does) translates the system calls to the network file system protocol understood by bns. Therefore, the constant namespace seen by the application is provided as a whole by bns. Figure 2 depicts the scenario described before in a Plan B system.

Thanks to its centralized architecture, the Octopus has a per-user namespace where all the resources can be looked up and accessed. We have found that having the same resource always at the same path (*i.e.*, having a global namespace and a set of conventions) is essential to keep the complexity contained. The global namespace serves various functions. It provides a constant environment where programs can live. For example the printing service for the current machine is always found in the same point of the namespace (a path in the file system), so any program that needs to print a file can simply use it. The global namespace also provides a way to find resources in the network. In the Octopus, the global namespace contains a placeholder exporting the namespace as seen by each machine. If a user wants to access a resource from a remote machine, she only has to open the directory representing that machine and browse it.

This *simple* approach actually makes the user believe that she is always seeing the same virtual computer through different view ports. At the same time, it abstracts the complexity for the programs using the machine.

The counterpart of bns in the Octopus is mux. Mux is a userspace program which is a *proxy* file server. It just redirects the file system operations, by translating them from the network file system protocol messages to the operating system operations (*i.e.*, system calls). This component is instantiated for each resource class in the system. Each instance knows the mount points for several concrete resources of its class and forwards the operations accordingly. The specific resource selected depends on the context information, the available instances and users specification [3].

**Figure 3.**
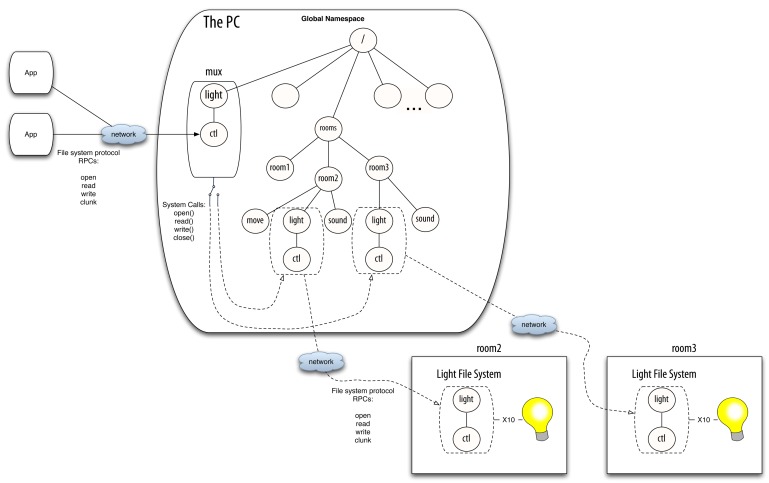
An example scenario with mux.

For example, suppose that a client wants to get or modify the state of a light co-located with the user. Suppose that this device is represented by a single virtual file, ctl, which provides a string with the state of the light when read, and changes the state when written. To accomplish this task, the device can drive any kind of binary output (e.g., X10 or KNX). There can be several light switches in the smart building, mounted in their specific mount points in the global namespace (e.g., the name of the directories can be the numbers of the offices, or follow any other convention). Figure 3 depicts a scenario with three rooms, with different sensors and actuators installed. Be aware that the different sensors and actuators (e.g., the binary outputs) that mux commutes between do not need to belong to the same technology. They only require to be abstracted as virtual file systems following the same conventions in their interfaces.

Mux is able to select the corresponding file and redirect/translate the client petitions according to the context data provided by the system (*i.e.*, the physical location of the user and the lights). When the client sends a message to open and read/write the file, mux performs the corresponding system calls to read/write the specific file. When the user moves to another room and the context data changes, mux tries to select a new binary output located in the new physical location of the user by looking it up in a central registry. The client can continue accessing the files provided by mux, unaware of this reconfiguration. The only detail that needs to be dealt with is that the files already opened since before the reconfiguration will return an error. The client has to deal with the error accordingly, like properly written programs always do. The program can retry on error, or open and close the file for each operation, which is what is normally done by shell scripts. Returning this error is important in case the client needs to react to the reconfiguration.

The main difference between these two schemes is that bns is much more complex than mux. First, bns includes all the required machinery for dealing with more than one resource at the same time, whereas there is an instance of mux per multiplexed resource. Second, bns has to maintain the correspondence between the elements of the file system protocol for both ends of the chain. While this gives the implementor freedom to alter the behavior of the multiplexer in interesting ways, it is more complex and error prone. On the other hand, mux translates the file system protocol messages to system calls, leveraging on the mechanisms provided by the kernel (e.g., the mount table).

## 3. Things That Worked Well

The central idea behind the Octopus is that the user perceives that the state she cares about (her session, the state of the applications and the services) is always available. Part of maintaining this illusion is having the user's session persist. This approach works particularly well. The user interface in the Octopus keeps its state on a central per-user service. As a consequence, the user can move from one computer to another in any place and continue where she left, with all the windows and user interface in the same layout. This persistence of the session makes the user believe she is all the time working in one computer and minimizes the time it takes to restart work.

As stated before, developing a new service for our systems involves programming a small userspace synthetic file server. Writing a file server may seem like a daunting task, but it is not more complex than, for example, implementing a web service (e.g., a RESTful service). Even further, most of the principles deemed as good practices by [5] (small known set of operations on named entities, *etc.*) are already part of the design of a file system.

A (synthetic) file system server must implement the service functions for the messages of the file system protocol. Note that the file system protocol we use (9P in Plan B, or Op in the Octopus) is specially designed with this task in mind and it is of a high level of abstraction, in contrast to other protocols like FUSE or NFS. There is a small number of messages, each representing a simple operation, like opening a file, traversing the file system tree, writing and reading data, *etc.* While a concrete server is tied to a concrete network file system protocol, adaptors can be interposed between the client and the server to use different protocols, best suited to the medium at hand. For example, the Octopus servers use 9P internally, Op on the network, and WebDAV on the native client side [6].

The hardest aspect of managing a file system is the shared tree data structure. The standard libraries provided by Inferno and Plan 9 help on this regard. In any case, most of the file servers we have implemented for exporting pervasive resources are one level, so they do not require dealing with this problem.

**Figure 4.**
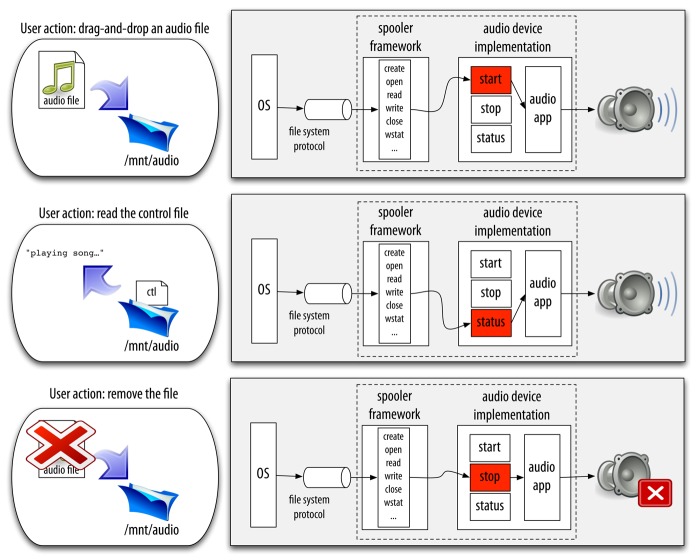
The simple scheme of a spooler based audio device in the Octopus.

In the Octopus, we even simplified the problem further for the most common pattern of interaction: a one level synthetic *active* directory, or *spooler*. All the scaffolding related to the management of the operations on the directory itself is just written once and reused as a framework. Then, specific spoolers can be specialized by providing operations for the files created within the directory they serve. These operations are executed when a file is created ( start) or removed ( stop). In addition, the spooler accepts specific commands through a control file. For example, this file is used to request the state of the operations (possibly performing the status operation for each of the created files). In the common case, the concurrency is managed by the spooler framework. The implementer of the specialization only has to deal with the operation running as a consequence of creating/removing the file. Figure 4 depicts an example audio service based on the spooler framework.

Of the services based on the spooler framework, we have found two especially useful, view and print. Both are extremely simple. They are based on the spooler framework. View displays any document copied into it and print sends it to the printer.

The reason for the success of these devices is twofold. The first and most important one is that the interface is of a *high level of abstraction* and utterly *simple*, which permits both users and programs to access it without problems. Principals can use them by dropping files in the directory using a file browser, or using *legacy* tools like the UNIX commands, or even saving a plain text file with an ancient text editor. Programs only have to use some standard system calls to create a new file in the directory, write it, and close it.

The second has to do with the implementation. These services are *available on all the operating systems* where we ran the Octopus daily: Mac OS X, Linux, Plan 9, or Windows. They are always at the same place (because of the global per-user namespace) and with the same interface (a spooler) so we use them spontaneously. Even further, the user interface of the Octopus has the view device integrated in it.

Another successful service we use is the voice device. This service is not based in the spooler. It is a simple one level file server: it serves only one file. This device synthesizes voice when text is written to this file. The reason we use it frequently is because it is integrated with the user interface/shell of the system. For example when a long running command (a command that takes more than 2 s to run) ends, the system uses the speech synthesis device to announce it. If the system speaks every time the user runs a command, it becomes annoying and obtrusive. On the other hand, if it speaks when a long running command ends, it is quite useful.

Among all the instances of these services available in the environment, the system needs to select the proper one. The decision of which one to select depends on the context. To provide the system with information of this context, we have the location framework. It is composed of a file system (where data about the physical location of the users and their machines is updated) and several programs and shell scripts we use to display that information through web pages and the user interface.

As source of data for the location framework we tried several sensors and devices (that the users needed to carry around) to sense which users were around, without success. The users would forget to carry their devices, or the sensors network would go down and would not be repaired. In the long term, the best solution we found and are still using today is sensing activity from the user on a terminal authenticated in her name. A service named idle provides such information. Terminals with a fixed location have this information hardcoded as part of their configuration. Mobile terminals retrieve their location as part of the logging process. Today this could be taken further with smartphones providing all sorts of location services. This strategy, combined with a clearing of the information if we have not heard from the user in a while (*i.e.*, have not sensed any activity on any terminal), has proven to work much better and to be more cost effective than any of the alternatives. As an example of the way we use this framework, there is a graphical application to display the users currently on-line on the system and their location using the location framework. It also uses the voice command when a user comes or goes to announce who is around, which has proved to be quite useful too.

## 4. Drawbacks and Pitfalls

Some services are better provided by a web site rather than by the system. Users tend not to be concerned where the data comes from as long as it is available at all times and easy to access and manipulate. This is especially true for content-rich services like music. If the user finds a service where she is relieved of the burden of taking care of the files, updating the metadata, looking up for new music files, *etc.*, this service will always take precedence. For example, this is the reason why Spotify, an on-line music streaming service, ends up being used more than the music device of the Octopus.

Another problem is maintaining the infrastructure. When analyzing AmI systems, deployment and maintenance costs are often forgotten or ignored. Some of the systems we have developed (ultrasound sensors, public displays) have ended up unused because of the costs of deployment and maintenance. It is utterly important that the systems update automatically, do not need configuration, and require very little infrastructure in their deployment. A system with much less precision and functionality will end up being used more if it requires less maintenance. A system requiring constant maintenance will be left unmaintained as soon as the users are busy elsewhere. New approaches minimizing infrastructure by either piggybacking on already present hardware, like WiFi access points [7], or cheap, widely available hardware like [8], are preferable to precise but expensive and hard to maintain infrastructures.

These costs are not only important in terms of money, but also in terms of how reliable the user perceives the service is. For example, if the user checks a public display and the information displayed is not accurate, she stops trusting it. As a consequence, the user will find alternative methods of finding that information.

Services which require the user to carry on some sensor are less likely to work, because the user will forget to carry it or the battery of the device will die. Unless the device is passive or has unlimited battery (or the battery lasts years) and it can be attached to a key or something that the user will care about, the service will end up being bypassed or simply ignored. Having the service as unobtrusive as possible is not another requirement; it is essential.

## 5. Lessons Learned

From the experience described above, we have distilled some general lessons. Even if they are not new, they are usually forgotten, especially in emerging fields like pervasive and AmI computing.

### 5.1. Keep It Simple

“Simplicity is the ultimate sophistication.”

Leonardo Da Vinci

Systems and applications are implemented over layers and layers of software. It is not rare to find out that different layers of software are providing the same services. In some occasions, complex frameworks or middleware are used just to access services provided by the operating system itself. In some recent joint project, we found out that a complex object oriented framework was being used just to use TCP sockets and accessing files in a UNIX system. Sometimes, learning to use this kind of framework correctly is at least as hard as learning to use the underlying system itself.

In our case, the previous experience developing and using systems software led us to create a new operating system. This system was provided with the mechanisms to enable pervasive computing environments, avoiding layers of complex and useless software. However, we made a mistake. We designed a complex architecture for Plan B.

As stated before, the Plan B architecture is Peer-to-Peer. The idea is that if some users got isolated from the network but still had connectivity between them, they could still interact. This added too much complexity to the system. This design requires extra machinery to support a highly dynamic decentralized scheme, and discover peers and services. And the worst part is that, in practice, the Peer-to-Peer architecture ends up not being useful.

First, user interaction at isolated places is uncommon. We made the assumption at that time that network partitions would be usual. In this scenario, only a Peer-to-Peer architecture can work. But this is not the common case. Most of the time, users are connected to the smart space infrastructure, maybe through poor WAN links or WiFi networks, but they can reach centralized services.

Second, and more important, in the end Plan B users depend on centralized services like data storage, authentication mechanisms, context infrastructure, CPU servers, and others. These services are single points of failure, no matter if the resources of the user are distributed following a dynamic Peer-to-Peer scheme.

Last, the complex Peer-to-Peer design led us to some incoherences in the namespace of peers while selecting resources available in the environment. For example, if there were several speech synthesis devices in a room, the different peers could select any of them, even using the same constraints and the same context information. In this case, the user could receive voice messages from different speech synthesis devices in the room. This situation could confuse the user.

The architecture of the Octopus is simpler. It uses some centralized services to control the rest of the system (e.g., the event delivery component). The resources of the user are distributed, but a central node controls them and manages a shared, global namespace for all the machines belonging to a user. This is the most simple and straightforward approach. This approach has worked well for powerful distributed systems like Google [9], and it also worked well for the Octopus.

There are two main problems associated traditionally with centralized services. The first one is scalability. Note that scalability in the Octopus approach does not pose a problem, because the central machine is per-user and the number of nodes belonging to a user is bounded. In other words, the system is naturally partitioned into manageable domains. The second problem is the latency introduced when accessing local devices or devices close to the local system, when the central node is far away. In the Octopus, local devices are directly accessed using the local namespace. Note that this is an optimization not part of the architecture itself, but trivial to implement. The problem of accessing a nearby service without traversing the central node is solved in the Octopus by a component called copy device. This device runs in each node and accepts copy requests for transferring remote data to a local destination, bypassing the central coordinator.

There is a trade-off between simplicity in the design and implementation provided by the centralized approach and the flexibility of the decentralized architecture. In our experience, the former is a clear winner, because the flexibility provided by the latter was not useful for us in practice, as explained before.

Simplicity is often forgotten when comparing different approaches, especially in the implementation, but it is paramount to take it into account. Defining a reduced set of features and separating policies from mechanisms also helps to maintain the system simple. This design principle, widely used for developing conventional systems, is also valid for pervasive computing systems and AmI environments. We have been very careful to separate policies (e.g., context-model, resource selection, *etc.*) from mechanisms (e.g., adaptation, reconfiguration, *etc.*).

### 5.2. Ancient Technology is Your Friend

“We have persistent objects, they're called files.”

Ken Thompson

Nowadays, some basic principles and technologies have been left behind. Simple approaches that worked well for previous systems are discarded just for the sake of innovation, and then they are reinvented or forgotten.

One example is the UNIX application model. This model is based on simple, specialized programs that can be composed in pipelines in order to perform complex tasks. This elegant and simple model proved to be successful for conventional operating systems. Nevertheless, current developers tend to design and implement huge all-in-one multipurpose applications [10]. The same occurs in pervasive computing. Sometimes, the whole smart space is just a huge application.

Another good example is XML. XML is widely used in pervasive computing systems, particularly after the recent increase in popularity of web services. Most of the times, XML is used just to describe a tree data structure, in which nodes are objects or data containers, for example in [11,12]. In other words, most of the times XML is used just to provide what the operating system already provides through a file system.

But a file system is much more than a tree description. It is comparable with XML and all the APIs, tools, and protocols to handle XML data.

A file server is a live program that dynamically processes requests over the objects it provides (*i.e.*, the files). Its interface is well-known and has been in use for decades. The operating system provides an API (*i.e.*, system calls) available for all programming languages, for free. It also provides a toolkit (*i.e.*, the shell) to manually or/and automatically manage these objects and hundreds of tools that can be combined to perform powerful tasks (*i.e.*, UNIX filters) over the objects, (un)serialize them (*i.e.*, compression commands), introspect the structure (e.g., listing commands), *etc.* This was invented decades ago.

Conventions in the use of devices are analogous to XML schemas. XML based interfaces and synthetic file systems are exposed to the same problems when their schemas and conventions are violated.

Files can be exported to the network. In order to do that, a file system protocol has to be used. Then, the user can perform read and write operations on the objects, that are represented by paths in the remote server. These read and write operations can be used to command actions or to retrieve stored or generated data. Many would think that we are describing a complex RESTful web service [5,13–15]. We are not. We are referring to network file systems, wildly predating them, and which also have a solid protection scheme, well-defined operations to create new objects and remove them, and have been in use for decades.

For example, it was trivial for us to write shell scripts to update our web pages and generate different versions of the contents of our location service, which is a file system. As part of a joint project with another university, we integrated their XML location framework into ours (some simple scripts and programs parsed the relevant information and updated our location framework). It is interesting to note that the other side of the project (*i.e.*, update the XML framework to include our space in theirs) was never done. It was not trivial to do so, in particular after considering security issues.

The most usual mistake is to argue that synthetic files do not provide types and/or type checking, for example, when used to execute commands or to exchange data represented as text. It may not seem so, but type-checking does not help much regarding correctness of the requests made and/or data retrieved. Note that clients and servers may be written in different programming languages. Some will be strongly typed, some not. Those that are typed may have different, incompatible, type systems.

Synthetic file servers must check data written for validity, like an OS kernel or a network server would. If the request made is invalid, an error is raised. It does not really matter in practice if the error is due to type checking or due to an invalid request. If the response given by a server is not correct, the client of the server is responsible for checking it for validity and acting accordingly. What we have seen in practice, if that when the user makes a mistake, the device raises an error, and the user tries again; this has never turned out to be a problem.

### 5.3. Mainstream Systems should be Integrated

“Anyone who has never made a mistake has never tried anything new.”

Albert Einstein

Pervasive services are not different from common operating system services. If you implement these services in your application, other applications will not be able to use them. Many AmI systems are just one big bundled application. Other approaches use middleware to provide the services to different applications. But middleware imposes to the programmer a development platform (a restricted set of programming languages, a concrete runtime, *etc.*). We decided to create a new operating system, Plan B.

Conventional systems, such as Mac OS X, Linux, or Windows were not able to take advantage of the Plan B components to run pervasive applications. This is a problem because we all need to use specific applications only available for such systems. Moreover, most people use these systems and are not ready to use a new operating system like Plan B. Of course, these conventional systems could follow the Plan B approach and implement its mechanisms to provide the same services to their applications. But this was not the case. In the end, we were using two different environments, the pervasive one, and the conventional desktop one.

We learned that conventional software and mainstream systems should be integrated with the pervasive services and the smart space.

The Octopus was designed to face this problem, which is why it is a hosted system. Moreover, it is able to provide its mechanisms to the host and its native applications, without using middleware. The applications running on the host are able to use the resources available in the Octopus global namespace as native system services, without using any middleware.

### 5.4. Latency is (Very) Harmful

“Nothing travels faster than the speed of light with the possible exception of bad news, which obeys its own special laws.”

Douglas Adams

Latency has a huge impact in the experience of the user: when it increases, interactive use of remote resources is not viable. Usability is tightly coupled with response time. Response time, in turn, is a function of the latency of the network in distributed systems. Of course, WANs have inherent high latency due to the long distances and congested routers. This is unlikely to improve [16]. In wireless and mobile networks, even if the distance between the client and the server is short and there are no intermediate hops, latency may become unbearable as a result of channel coding, retransmissions due to poor links, and accumulation of round-trips because of the protocol design.

Protocols performing a large number of synchronous requests and waiting for responses to perform actions over remote resources are undesirable, because the latency is accumulated for each request/response pair and the total latency of interactive actions is too high and annoying to the user. This was the case of the protocol used by Plan B (inherited from Plan 9 [17]).

The Octopus includes a new protocol, Op, designed to hide the impact of *latency* for interactive use. It reduces the number of messages to access remote resources [6]. Thanks to it we have been able to access local and remote services (including the user interface) with a file system interface successfully.

Intensive use of caches for the data of the user is also a good method to countermeasure the *latency* effects, but some resources cannot be cached (i.e., the devices). Caching must be done with extreme care. For example, documents can be cached, but the data generated by a camera cannot be retrieved from a cache.

Confining all I/O interaction to the client application can also reduce latency. This is possible when using high level interfaces to access remote resources, as described next.

### 5.5. High Level of Abstraction

“To those who do not know mathematics it is difficult to get across a real feeling as to the beauty, the deepest beauty, of nature.”

Richard Feynman

When developing both the Octopus and Plan B we have seen that interfaces having a higher level of abstraction behave better in terms of latency and they also end up being used more.

They behave better in terms of latency because they imply less operations being sent over the network. Compare sending all the interaction of the user (mouse clicks, image updates, *etc.*, like VNC [18] does) with sending a high level view of the widgets state (like the user interface management systems of Plan B and the Octopus do). The difference between them is that one is providing a lower level of abstraction, almost at the level of the devices, whereas the other is doing the opposite, exporting widgets. Because of this, there is no need to *micromanage* the interface remotely, which would imply more messages being exchanged and the latency of each of these operations added. High level abstractions end up being used more because they are easier to understand, use, and program. An example of this is the print or the view interface in the Octopus. These interfaces are so simple (leave this file in a specific directory and it will be printed or viewed) that it is trivial to use and program them. We use them from scripts and the user interface constantly.

Another lesson to be learned is that the data exchanged and provided through interfaces needs to be in a standard common format whenever possible. For example, when providing a music interface, it is better to provide a device that can understand MP3 than some not commonly known PCM audio format. A possible consequence is that the interface is specialized for the task. An interface suitable for playing music will not be suitable for real time audio generation. There is a trade-off between generality of use and specialization, but it pays off in terms of usability.

This should not be confused with hiding error details and making the errors transparent for the applications. Applications must be able to detect errors and try to recover.

## 6. Related Work

Related work is too extensive and varied. A lengthy discussion can be found in previous publications [1–4]. In this section we will focus on the key differences between our systems and other systems found in the literature.

Plan B and the Octopus are aimed at enabling personal pervasive environments in the Internet. In this respect, they are different from other systems designed to build smart spaces, such as smart rooms or intelligent buildings (e.g., Interactive Work Spaces [19]).

Our systems differ mainly from other systems in that they provide system support for pervasive applications by using (synthetic) files as the main interface. This way, the pervasive services are provided at the OS level (*i.e.*, at the file system level). Most systems found in the literature provide system support for pervasive applications by using middleware [19–23]. For example, GAIA [20] is based on a middleware infrastructure that offers the services to create pervasive applications (context service, event delivery, *etc.*). Another example is iROS [19], the middleware of the Interactive Work Spaces, which allows dynamic loose coupling between the components of the smart space.

In general, middleware based systems do not allow to integrate legacy software with the new pervasive environment. Old applications have to be rewritten or wrapped to use the specific middleware. Moreover, the new pervasive applications have to be developed using particular languages, libraries, frameworks, or tools. On the other hand, Plan B and the Octopus provide the pervasive services directly at the OS level, so legacy applications are able to use the new resources without any modification. Moreover, brand new pervasive applications are not tied to any particular development technology (they only have to use the native OS interface to access files, which is supported by most languages).

Multiple web architectures have been proposed to support pervasive applications [13–15,24]. SOAP based web services are complex and hard to compose [14]. Lately, a lot of research has been going into developing and composing RESTful web services [5] for pervasive computing [13–15]. RESTful services are viewed as resources and can be identified by their URLs. These resources are accessed through a restricted set of operations, mainly to read and write them. In this sense, they are similar to resources represented by a network file system. This architectural approach is relatively recent. In contrast, network file system technology is mature. File systems provide security (authentication and access control), resource organization (the file system is a browsable, dynamic hierarchy *per se*), and a powerful set of programmatic tools (shells, UNIX commands, synchronization and replication tools, *etc.*). It is not clear how to solve these issues when using web services (specially the security issues[14,25]).

## 7. Conclusions and Open Issues

In this paper we describe our experiences designing, developing, and using two operating systems for providing system support for pervasive applications, Plan B and the Octopus. We describe their mechanisms for dynamic reconfiguration and some lessons we have learned, related to simplicity, useless innovation, compatibility, network limitations, and abstraction level. Even though these lessons are general in systems software programming, they have been widely ignored in the fields of pervasive computing and AmI environments. Finally, we present the main differences between our systems and other pervasive computing systems.

Although the Octopus solved several issues we had with the previous system, Plan B, there are still open issues needing further research.

One of the basic assumptions of our systems (centralized like the Octopus or Peer-to-Peer like Plan B) is that they are always connected. This assumption has proven to be tricky. Setting aside the latency problem we already talked about, networks are not as reliable as we thought. In many instances, the network comes and goes randomly, for different reasons. The assumption that “the user is always connected” needs to be changed to “the user is connected most of the time”. This change implies that the system needs to support at least transient network disconnections and network roaming.

Even further, it would be interesting, even if the architecture is still centralized, to have some form of disconnected operation, which lets the user to continue working and goes back to synchronous behavior when the network comes back. The local machine could be viewed as a cache of the central system, which maintains coherency when possible, taking an approach similar to Coda [26] and Odyssey [27].

Related to this is automatic replica management, where a replica of the system is maintained close to the user and maintained coherent with other replicas around.

How to do all this (automated replicas, disconnected operation, *etc.*) automatically and without the user intervention is part of the work we will be doing for our next system.
